# Navigating the Eukaryotic Paradox: Insights for AI-Driven Computational Strategies Against Antifungal Resistance

**DOI:** 10.3390/microorganisms14102380

**Published:** 2026-10-17

**Authors:** Vivian Tullio

**Affiliations:** Department of Public Health and Pediatrics, Microbiology Division, University of Turin, 10126 Turin, Italy; vivian.tullio@unito.it

**Keywords:** emerging fungal pathogens, computational strategies, antifungal resistance, AI drug repurposing, medical mycology, eukaryotic paradox, biophysical barriers, deep learning, biofilms

## Abstract

The escalating crisis of invasive fungal infections and increasing antifungal resistance demand rapid therapeutic innovation. Given the critically narrow clinical arsenal, artificial intelligence (AI) and machine learning (ML) have emerged as pivotal strategies to accelerate drug repurposing, providing a rapid alternative to *de novo* drug discovery. However, translational success in medical mycology faces a major biological hurdle: the “eukaryotic paradox,” the extensive genomic, metabolic, and structural homology between fungal cells and human hosts. This review provides a critical and comparative analysis of AI-driven drug repurposing as a therapeutic strategy. This review characterizes the hierarchies of structural barriers, biophysical transport bottlenecks, and computational failure modes across bacterial, viral, and fungal pathogens to explain why medical mycology remains one of the most difficult frontiers in computational pharmacology. It reviews limitations of fungal cell walls, biofilm EPS matrices, morphologic dynamic dimorphism, and scarcity of high-resolution 3D protein structures for AI models. Next-generation computational approaches and experimental platforms, ranging from physics-informed neural networks and transcriptomic profiling to microfluidic organ-on-a-chip validation systems, are also described to overcome these resistance mechanisms. Finally, this review emphasizes the importance of the development of tailored, physics-informed AI paradigms for effective therapeutic strategies against emerging resistant fungal pathogens.

## 1. Introduction

Invasive fungal infections (IFIs) account for over 1.5 million deaths annually worldwide. Climate change and shifts in global human migration are accelerating the geographic range and thermal tolerance of opportunistic fungal species [1]. Consequently, the rapid identification and deployment of effective therapeutic strategies have become an imperative global health priority.

However, the traditional pipeline for *de novo* drug discovery is notoriously slow, prohibitively expensive, and fraught with high attrition rates. Bringing a single novel chemical entity to market typically requires over a decade and financial investments of approximately billions of dollars [2]. When confronting rapidly evolving drug-resistant pathogens, time represents a critical limiting factor. To escape this bottleneck, Artificial Intelligence (AI) is transforming drug repurposing, a computational strategy that identifies novel therapeutic indications for previously approved or investigational molecules.

Because these drugs possess well-characterized toxicological profiles, proven human tolerability, and documented pharmacokinetics, they can bypass early-stage clinical safety trials (Phase I), drastically reducing development timelines and associated financial expenditures [3]. Today, AI-driven computational methodologies are fundamentally redefining how we combat bacterial, viral, and fungal threats. While AI computational frameworks were initially benchmarked against bacterial and viral targets, applying these models to eukaryotic fungal pathogens introduces unprecedented biological and structural bottlenecks.

Unlike recent comprehensive scoping reviews on this topic [4], which systematically map the entire landscape of AI in fungal drug discovery using PRISMA-ScR guidelines, this manuscript is designed as a targeted narrative review. The objective is to provide a critical, mechanistic comparison of computational failure modes across specific pathogen classes. The literature search was conducted primarily in PubMed, Scopus, and Research Gate (2015–2026) using keywords such as “antifungal drug discovery” “artificial intelligence,” “drug repurposing,” “antifungal resistance,” and “computational pharmacology.” To frame this conceptual synthesis, the literature was selected based on relevance to computational drug repurposing, AI frameworks, and fungal resistance dynamics. Pre-2015 references were retained as foundational historical milestones that establish essential cell wall baselines and resistance mechanisms. For this comparative framework, this review is deliberately focused on bacteria, viruses, and fungi. Parasites were excluded from this specific analysis due to their extreme biological heterogeneity and the current lack of standardized, high-throughput screening datasets required for robust computational benchmarking, which contrasts with the more mature data ecosystems available for bacterial, viral, and fungal targets.

This paper describes the hierarchy of structural barriers, biophysical transport limits, and computational failure modes across bacterial, viral, and fungal pathogens to clarify why medical mycology is one of the most problematic frontiers in computational pharmacology and drug repurposing against antifungal resistance.

A comprehensive comparative summary of the barrier hierarchies is provided in Table 1.

## 2. The Antibacterial Front: Combating Superbugs and Physical Permeation Barriers

Antimicrobial resistance (AMR) represents one of the most pressing public health crises of the twenty-first century. As bacteria rapidly evolve mechanisms to evade our current clinical arsenal, the pipeline for novel antibiotic classes has virtually dried up, threatening a return to a pre-antibiotic era [5]. Advanced machine learning (ML) and deep learning (DL) architectures are increasingly deployed to screen vast chemogenomic libraries of non-antibiotic drugs, synthetic compounds, and natural products to uncover hidden bactericidal or bacteriostatic properties [6]. A landmark example was the deployment of deep neural networks (*Artificial Neural Networks*, ANNs) to identify halicin, a molecule originally investigated as an antidiabetic agent, as a potent broad-spectrum antibiotic [6,7]. ML models evaluate the molecular fingerprints of thousands of existing compounds to predict their binding affinity to conserved bacterial targets, such as those involved in cell wall biosynthesis or ribosomal translation. Furthermore, AI excels at identifying synergistic drug combinations: by pairing a conventional antibiotic with a repurposed non-antibiotic agent, AI can disarm resistance mechanisms, effectively restoring the antibiotic’s original potency.

Despite these computational successes, data limitations, physical transport barriers, and translational hurdles present significant challenges to translating in silico screening into clinical efficacy. ML models are inherently limited by the quality of their training data. Current datasets for antibiotic discovery face severe systemic limitations, primarily due to a pronounced publication bias favoring positive outcomes. Because peer-reviewed literature predominantly highlights successful experimental results, decades of negative data, including molecules that demonstrated little or no antimicrobial efficacy, are often unpublished or kept behind proprietary barriers [8]. Deprived of these negative training exemplars, AI models suffer from elevated false-positive rates and fail to accurately map the chemical boundaries of non-efficacy. This problem is further compounded by inter-laboratory heterogeneity across historical screening data compiled over the past half-century. Widespread variations in assay protocols, bacterial strain selection, and minimum inhibitory concentration (MIC) endpoint definitions introduce profound noise into ML pipelines, ultimately compromising model generalizability across independent biological contexts.

The most critical clinical threats reside within Gram-negative pathogens, such as *Acinetobacter baumannii* and *Pseudomonas aeruginosa*, which present severe biophysical barriers to AI-driven drug discovery [9,10]. This challenge stems primarily from a marked disconnect between in silico predictions and in vivo efficacy. While an algorithm may successfully identify a candidate molecule that perfectly inhibits a vital bacterial target within a computational simulation, current models struggle to accurately predict outer membrane permeation kinetics across the asymmetrical, bilayered envelope characteristic of these microorganisms [9]. Furthermore, even when a compound successfully traverses this selective macromolecular barrier, computational pipelines frequently fail to anticipate whether the molecule will be immediately extruded by promiscuous bacterial efflux pumps prior to reaching its intracellular target [11].

Compounding these biophysical obstacles are regulatory, toxicological, and macroeconomic constraints that hinder real-world application. Deep learning architectures, particularly ANNs, are notoriously opaque. Operating as “black boxes,” these computational models generate high-probability predictions for antimicrobial activity without elucidating the underlying molecular mechanisms of action [12]. For instance, an algorithm might predict with 95% statistical confidence that an established antihypertensive agent will exert bactericidal activity against a specific pathogen, yet remain incapable of explaining the structural or biochemical basis of this inhibition. This mechanistic opacity presents a significant hurdle for regulatory authorities such as the US FDA or EMA, which remain hesitant to grant approval, even for repurposed molecules, in the absence of a defined mechanism. This regulatory hurdle is further complicated by the safety paradox of escalated dosing: while a repurposed candidate may possess a well-documented clinical safety profile at its approved therapeutic indication, clearing a systemic, acute bacterial infection often requires serum concentrations far exceeding original physiological baselines. Current AI models frequently fail to anticipate the off-target toxicity and organ strain triggered by such high-dose regimens, highlighting a critical gap between computational predictions of efficacy and real-world clinical viability. Finally, market failures severely disincentivize antibiotic commercialization, as antimicrobial stewardship frameworks mandate that effective new agents be held in reserve, limiting sales volumes and preventing developers from recouping clinical trial expenditures [13]. While Gram-negative bacterial envelopes present complex permeation barriers, the computational challenges escalate significantly when transitioning from prokaryotic cell walls to eukaryotic fungal structures.

## 3. The Antiviral Front: Rapid Pandemic Response and Host Physiology

Unlike bacteria, viruses are obligate intracellular parasites with minimal autonomous metabolic machinery, replicating entirely by hijacking host cellular pathways. Consequently, AI-driven antiviral discovery encounters distinct computational and biological hurdles. Viral outbreaks necessitate rapid containment strategies that traditional, *de novo* drug pipelines cannot accommodate [3]. AI-based computational repurposing has thus emerged as a cornerstone of rapid-response pharmacology. Using deep learning models, algorithms can rapidly evaluate the binding affinities of thousands of small molecules against predicted 3D structures of novel viral proteins within days [14]. Moreover, because viruses depend on the host, AI leverages network pharmacology to map host–pathogen interaction networks, prioritizing host-directed therapeutics that target host factors essential for viral replication [15]. By targeting host proteins rather than hyper-mutable viral enzymes, host-directed approaches establish a higher genetic barrier to resistance, remaining effective across divergent viral strains.

The application of AI to antiviral drug discovery faces severe biological and computational constraints, primary among which is the disconnect between dynamic mutational landscapes and static structural modeling. RNA viruses, such as influenza, HIV, and coronaviruses, exhibit exceptionally rapid evolutionary kinetics [16]. Because AI models are conventionally trained on static crystallographic snapshots of viral proteins, even single amino acid substitutions within a target-binding pocket can rapidly invalidate an AI-predicted ligand’s efficacy [14]. Furthermore, current computational frameworks possess limited fidelity in forecasting viral evolutionary trajectories or predicting prospective escape mutations under selective drug pressure.

This challenge is further compounded by the “host-dependence barrier” inherent to obligate intracellular parasites. Because viral replication is inextricably linked with host cell physiology, targeting viral pathways carries a high intrinsic risk of cellular disruption [15].

These predictive limitations are exacerbated during novel outbreaks by the data scarcity paradox of emerging pathogens. Deep learning architectures rely heavily on extensive historical datasets; consequently, upon the emergence of a novel pathogen with pandemic potential, computational pipelines encounter an acute deficit of empirical data [3]. Direct extrapolation from established viral families remains highly uncertain due to subtle yet functionally consequential genomic variations.

Even when potential candidates are identified, pharmacokinetic and pharmacodynamic disconnects frequently hinder clinical translation. Antiviral efficacy typically hinges on administration within a narrow 24 to 72-h physiological window post-infection, yet binding-focused AI models rarely incorporate these rigid in vivo time constraints. Moreover, phenotypic screens routinely produce a high frequency of in vitro false positives, flagging diverse chemical classes (e.g., antimalarials or antipsychotics) that suppress viral replication through non-specific pathways like lysosomal pH alteration [17]. Translating these in vitro effects in vivo frequently demands cytotoxic or physiologically unachievable serum concentrations.

Finally, viruses exhibit remarkable structural variety in contrast to bacteria, which have conserved targets such as peptidoglycan cell walls. A single, cohesive antiviral AI architecture cannot be developed because of the lack of universal viral target architectures, necessitating customized pipelines for various viral families. Targeting host–pathogen interactions and host-directed antiviral methods are conceptually similar, but fungi provide a far more difficult problem because of their autonomous eukaryotic cell biology and proteome homology with human hosts.

## 4. The Antifungal Front: Addressing Eukaryotic Complexity, Biofilm Shielding, and Computational Strategies Against Antifungal Resistance

The application of AI to antifungal drug discovery presents the most demanding computational and biological challenges in modern pharmacology. While invasive fungal infections caused by opportunistic pathogens such as *Candida auris*, *Aspergillus fumigatus*, and *Cryptococcus neoformans* carry alarming mortality rates exceeding 50% in immunocompromised populations, medical mycology remains historically under-funded and under-researched compared to bacteriology and virology [18,19]. The central impediment to designing therapeutic strategies is fundamental to cell biology: fungi are eukaryotic organisms that share extensive genomic conservation, metabolic pathways, and structural homology with human host cells [20,21]. Consequently, identifying repurposed small molecules that selectively exert fungicidal activity without triggering catastrophic off-target host toxicity represents an enormous computational optimization challenge [22,23]. This methodological bottleneck directly contributes to the current clinical reality, where management remains constrained by a narrow therapeutic arsenal historically dominated by three primary classes: polyenes, azoles, and echinocandins, alongside pyrimidine analogs (flucytosine) and allylamines (terbinafine) [19,20]. Although novel classes, including echinocandin derivatives (rezafungin), triterpenoids (ibrexafungerp), and orotomides (olorofim), enhance this pipeline, the diversity of structural targets remains critically limited [19,20]. Current standard-of-care antifungals exploit essential fungal-specific pathway components, such as ergosterol biosynthesis and beta-(1,3)-D-glucan synthase. However, structural conservation in off-target human enzymes (e.g., human cytochrome P450 enzymes interactable by azoles) drives clinically significant hepatotoxicity and adverse drug–drug interactions [18,19,21].

This **eukaryotic paradox** severely complicates AI-driven screening pipelines. Because essential fungal enzymes (such as Hsp90 chaperones, calcineurin, or cytochrome P450 enzymes involved in sterol synthesis) closely mirror their human orthologues, virtual screening algorithms tasked with searching non-antifungal drug libraries encounter extreme selectivity barriers [24]. Standard molecular docking and deep learning scoring functions often fail to discriminate between subtle active site variations between fungal enzymes and their human homologues. As a result, candidate repurposed molecules identified in silico often demonstrate significant cross-reactivity with human targets, leading to serious clinical issues such as hepatotoxicity, cardiotoxicity, or nephrotoxicity [24]. This clinical challenge has historically been exacerbated by a severe scarcity of experimentally resolved target fungal structures, with very few available as ligand-bound complexes in the Protein Data Bank (PDB) [25]. While deep learning tools such as AlphaFold and ESMFold have dramatically expanded structural coverage across fungal proteomes (>98% predicted proteomic coverage) [26], these models represent computational hypotheses that ultimately require rigorous experimental validation (e.g., X-ray crystallography, cryo-EM, or biophysical assays).

Since deep learning architectures require high-resolution empirical data to accurately model complex drug–target interactions, the structural underrepresentation of pathogenic fungi creates a major bottleneck in training datasets for supervised machine learning and structure-based drug design [25,26].

Consequently, modeling dynamic conformational changes and binding affinities remains exceptionally difficult. Furthermore, historical empirical data regarding fungal replication kinetics, membrane lipid composition (such as ergosterol vs. cholesterol dynamics), and phenotypic responses remain fragmented, unstandardized, and isolated across non-interoperable databases [20].

Recent advances indicate that integrated computational approaches can effectively address these difficulties. Chai et al. [27] successfully predicted Dp44mT, a previously known compound, as a novel anti-*C. albicans* agent by employing an ensemble machine learning framework along with multi-omics strategies. This compound alters cellular iron homeostasis and effectively circumvents traditional azole resistance mechanisms [27]. A recent work by Li et al. [28] modeled the 3D structure of the enzyme *Af*DHODH and analyzed existing inhibitor mechanisms using molecular docking, dynamics simulations, and binding energy calculations. Virtual screening of the ZINC20 and ChEMBL databases led to the discovery of 13 candidates, from which in vitro assays identified two novel micromolar inhibitors (IC50 < 100µM) with distinct scaffolds, demonstrating the efficacy of homology modeling in targeted antifungal drug discovery.

In addition, to overcome current limitations in peptide novelty and diversity, recent work by Wang et al. [29] utilized a generative AI latent diffusion model for de novo antimicrobial peptide (AMP) design. This computational approach successfully yielded 25 functional antifungal peptides from 40 synthesized sequences, highlighting AMP-29 for its selective efficacy against *C. glabrata* and validated performance in a murine infection model.

Beyond target homology, severe biophysical and structural barriers disconnect in silico binding predictions from in vivo therapeutic clearance. The fungal cell wall is a dynamic, highly cross-linked macromolecular matrix composed of chitin, beta-(1,3)-glucans, beta-(1,6)-glucans, and outer mannoproteins [30]. This rigid structure poses an immense mechanical and steric permeation barrier to high-molecular-weight compounds. While molecular docking algorithms successfully predict ligand-binding kinetics at isolated receptor sites, current AI models lack the biophysical parameters needed to simulate molecular diffusion dynamics through this complex carbohydrate mesh.

This transport bottleneck is substantially amplified when fungal pathogens transition into refractory biofilm communities attached to mucosal surfaces or medical devices, significantly driving clinical antifungal resistance [31,32]. Fungal biofilms synthesize an extracellular polymeric substance (EPS) matrix rich in exopolysaccharides, extracellular DNA, lipids, and proteins. This matrix acts as a physical shield, trapping repurposed drug molecules via charge interactions or enzymatic degradation before they can reach cell-surface targets. Furthermore, cells deep within the biofilm matrix enter a metabolically dormant “persister” state, rendering target-binding algorithms ineffective since these persister cells downregulate metabolic targets targeted by standard AI models. Standard high-throughput virtual screening models evaluate isolated unicellular states in suspension, completely failing to account for physical shielding, persister cell physiology, and altered drug accumulation within resilient biofilm architecture. To yield clinically actionable drug repurposing strategies, AI computational models must evolve beyond raw binding-affinity predictions to integrate physical diffusion dynamics across EPS matrices.

Compounding these structural challenges are dynamic **morphogenetic transitions** and an excessively restricted baseline chemical space. Many lethal fungal species exhibit morphological dimorphism, switching dynamically between unicellular yeast phases, pseudohyphae, and invasive, filamentous hyphal structures in response to host environmental cues such as body temperature, ambient CO_2_ concentration, or local pH shifts [33]. This morphological transition fundamentally reshapes the surface proteome, cell wall structure, and transcriptomic landscape of the fungus. An AI model trained on static yeast-phase data will predict ligand efficacy against targets that may be completely downregulated or physically masked during invasive hyphal growth in tissue.

Finally, because systemic antifungal therapy relies almost entirely on azoles, polyenes, and echinocandins, the baseline training datasets for machine learning models lack chemical diversity. This narrow structural baseline deprives algorithms of broad “chemical intuition,” making non-antifungal drug repurposing predictions especially prone to false positives and biological failure during laboratory validation.

## 5. Methodological Pillars of AI Integration

Despite these domain-specific biological and computational constraints, the transformative potential of AI in cross-disciplinary drug repurposing is anchored in three primary methodological paradigms. These technological pillars collectively enable researchers to bypass conventional, empirical discovery routes by systematically interrogating pathogen vulnerability, host response dynamics, and the broader biomedical literature.

The **first fundamental pillar** unites structural biology with deep learning architectures. Deep neural network frameworks, exemplified by milestone developments such as AlphaFold, can predict the tertiary and quaternary 3D structures of pathogen proteins with near-atomic precision directly from primary amino acid sequences [34]. This computational breakthrough overcomes historical bottlenecks caused by the slow, labor-intensive process of crystallizing difficult-to-resolve target proteins. By generating high-fidelity structural models for uncharacterized pathogen targets, these systems facilitate high-throughput virtual screening and molecular docking simulations. Algorithms rapidly evaluate millions of existing small-molecule ligands against virtual binding pockets, scoring spatial fit, conformational adaptability, and binding free energies to prioritize candidates with high receptor affinity. In medical mycology, this pillar is particularly instrumental for modelling fungal-specific targets, such as predicting the 3D conformational changes in *A. flavus* Cyp51A to elucidate azole resistance mechanisms or mapping the structural epitopes of *Aspergillus* surface proteins [35].

The **second core paradigm** focuses on transcriptomic profile matching to target systemic host–pathogen interactions. Rather than focusing strictly on direct pathogen inhibition, this approach uses machine learning to decode the complex host transcriptomic perturbations induced by infection. Algorithms map high-dimensional gene expression signatures, capturing upregulated and downregulated host pathways during disease states, and cross-reference them against extensive perturbation databases, such as the Connectivity Map (CMap) [36]. By identifying small molecules that induce anti-correlated expression profiles, AI can predict repurposed therapeutics capable of reversing pathogenic gene signatures or bolstering host cellular defenses, effectively dampening infection-induced pathology. For fungal pathogens, this paradigm is highly valuable for identifying compounds that reverse the transcriptomic signatures associated with morphological dimorphism, such as the yeast-to-hypha transition in *C. albicans*, thereby blocking tissue invasion [29]. By learning the latent sequence-function space of membrane-active peptides, these models generate structurally diverse candidates with enhanced antifungal potency, reduced hemolytic toxicity, and targeted fungal cell membrane disruption.

**The third methodological pillar** leverages biomedical Natural Language Processing (NLP) to mine latent unstructured knowledge across vast scientific ecosystems. Large-scale transformer-based models take in and combine millions of peer-reviewed articles, clinical trial registries, chemical patent databases, and electronic health records [37]. Through advanced named entity recognition (NER) and knowledge graph construction, NLP models extract hidden phenotypic correlations, off-target interactions, and secondary clinical outcomes that have remained buried within disconnected literature. By connecting disparate empirical findings across disciplines, NLP tools surface non-obvious mechanistic links between established drugs and pathogen susceptibility that would otherwise elude manual human review. Crucially, these NLP frameworks can also mobilize knowledge regarding non-conventional or historically sidelined therapeutic agents, such as repurposed antiparasitics (e.g., ivermectin) [38] or medium-chain fatty acids, by uncovering hidden antifungal properties buried in disparate, non-mycological literature.

This approach provides fine-grained thermodynamic resolution required to evaluate resistance-conferring point mutations in key fungal enzymes (e.g., *Af*DHODH and CYP51), enabling the rational optimization of leads capable of overcoming kinetic resistance mechanisms [28].

## 6. Emerging Paradigms and Next-Generation Solutions

To bridge the persistent gap between in silico predictions and real-world clinical translation, the field is rapidly advancing toward next-generation paradigms that directly address the underlying physics, physiological complexity, genomic diversity, safety profiles, and ethical governance of AI-driven drug discovery. Traditional deep learning architectures rely exclusively on statistical pattern recognition from historical training sets, often neglecting fundamental physical transport constraints. To resolve this, researchers are deploying Physics-Informed Neural Networks (PINNs), which directly embed thermodynamic principles, electrostatic dynamics, and molecular diffusion equations into the network’s loss functions. This approach improves predictions of compound permeation across the Gram-negative outer membrane or fungal chitin walls while reducing black-box opacity. PINNs have been proposed as a computational paradigm to model reaction-diffusion kinetics through the complex extracellular polymeric substance (EPS) matrix of fungal biofilms [39,40,41]. Concurrently, to mitigate the high frequency of in vitro false positives originating from static 2D cell cultures, computational virtual screening is being systematically coupled with biomimetic microfluidic organ-on-a-chip technologies. These platforms replicate dynamic fluid flow, physiological shear stress, tissue-tissue interfaces, and active biofilm formation, enabling high-throughput automated validation of repurposed drugs within biomimetic infection microenvironments. [42].

Furthermore, acknowledging that historical screening pipelines relied heavily on standardized laboratory reference strains, modern computational strategies increasingly integrate population-scale pangenomics to evaluate target conservation across the fluid accessory genome [43]. Concurrently, deep learning-driven structural modeling enables high-throughput prediction of three-dimensional target conformations, facilitating molecular docking and binding affinity evaluations [43]. For instance, machine learning models trained on genomic variants within *ERG11*, *FKS1*, and *TAC1* effectively map structural perturbations that confer resistance to azoles and echinocandins in clinical isolates of *C. auris* and *C. albicans*. The integration of computational chemistry with systems biology has been recognized as a powerful strategy for accelerating early-stage drug discovery [44,45].

This ensures that AI-selected molecules maintain broad efficacy against highly diverse clinical isolates and emerging resistant lineages rather than single reference variants [45]. To prevent late-stage clinical attrition driven by off-target toxicities or cytotoxic dosing requirements, contemporary pipelines are also implementing early-stage multi-objective ADMET profiling, evaluating target binding affinity in parallel with predictive models for cardiac toxicity (e.g., hERG channel inhibition), hepatic clearance, and cytochrome P450 interactions during the initial virtual screening phase [46]. Finally, as generative AI architectures and active-learning platforms gain unprecedented capabilities in predicting molecular bioactivity and toxicity, the scientific community is establishing responsible AI governance and biosecurity guardrails to proactively manage dual-use risks. This is particularly critical in infectious research, where synthetic biology frameworks must be coupled with biosecurity guidelines to prevent the accidental or deliberate engineering of virulent or immune-evading pathogens [46,47]. Addressing these biosecurity challenges requires oversight mechanisms to ensure that advanced computational frameworks cannot be exploited to design biological toxins or bypass existing antifungal defenses [48].

## 7. Comparative Synthesis Across Pathogen Classes

To systematically navigate this multi-kingdom landscape, computational pipelines must account for the distinct biophysical constraints, delivery bottlenecks, and computational limitations imposed by each pathogen class. A comparative overview of these structural hierarchies is summarized in Table 1.

## 8. Concluding Remarks

The integration of AI into drug repurposing constitutes a paradigm shift in modern pharmacology and strategy against infectious diseases. Nevertheless, computational algorithms represent merely an initial predictive framework. To successfully mitigate the global burden of emerging resistant fungal pathogens and overcome clinical resistance mechanisms, in silico models must be seamlessly coupled with biomimetic validation platforms and rigorous clinical stewardship, thereby reconciling digital abstractions with the fundamental complexities of fungal physiology.

Moving forward, three specific, actionable research priorities to accelerate antifungal drug repurposing are proposed. First, the scientific community must establish standardized, high-quality benchmark datasets specifically curated for fungal phenotypic screening, including negative data, to train more robust and generalizable machine learning models. Second, priority must be given to identifying and validating novel, fungal-specific molecular targets that lack human orthologues, particularly those involved in biofilm matrix synthesis and morphological dimorphism. Third, integrating multi-omics data with physics-informed AI models are essential to accurately predict drug permeation through the complex fungal cell wall and EPS matrices, bridging the gap between target binding and actual intracellular accumulation.

Finally, to refine the computational approaches, it is also crucial to address fundamental challenges in pathogen characterization across all classes.

For instance, in virology, the definition of novel viral variants increasingly relies on PCR amplification rather than the actual isolation of infectious particles. This practice does not represent the actual isolation of a virus as defined by the traditional Rivers criteria to confirm an infectious agent, prompting a necessary re-evaluation of these foundational postulates in the genomic era [49].

Ultimately, bridging the gap between computational prediction and clinical reality requires not only advanced algorithms but also a rigorous, standardized biological foundation.

## Figures and Tables

**Table 1:** Pathogen Barrier Hierarchies in Antimicrobial Drug Discovery: Structural classification of cellular and extracellular barriers across major pathogen classes.

Pathogen	Primary Structural Barrier	Secondary/ External Barrier	Key Biophysical Challenge for Drug Delivery	Primary AI Computational Limitation
Bacteria	Peptidoglycan cell wall	Asymmetrical lipid outer membrane (Gram-negative), biofilm exopolysaccharides (EPS)	Selectivity across lipid/murein layers & efflux pump extrusion	Inability to predict membrane permeability & explain MoA ** (“Black Box”)
Viruses	Protein capsid	Lipid envelope (enveloped viruses)	Encapsulation stability & membrane fusion requirements	Failure to simulate hyper-mutation rates & host off-target toxicity
Fungi	Rigid chitin & * Beta-glucan matrix	Dense extracellular biofilm exopolysaccharides (EPS)	High molecular weight penetration barrier & biofilm matrix resistance	Scarcity of experimentally resolved, ligand-bound PDB *** structures for AI training & challenges in discriminating subtle structural homology modeling

* & = and; ** MoA = mechanism of action; *** = PDB = Protein Data Bank (the primary global database archiving 3D structural data of biological macromolecules obtained through experimental methods such as X-ray crystallography, cryo-electron microscopy, and NMR spectroscopy).

## Data Availability

No new data were created or analyzed in this study. Data sharing is not applicable to this article.

## References

[1] Denning D.W. (2024). Global incidence and mortality of severe fungal disease. Lancet Infect. Dis..

[2] Wouters O.J., McKee M., Luyten J. (2020). Estimated Research and Development Investment Needed to Bring a New Medicine to Market, 2009–2018. JAMA.

[3] Pushpakom S., Iorio F., Eyers P.A., Escott K.J., Hopper S., Wells A., Doig A., Guilliams T., Latimer J., McNamee C. (2019). Drug repurposing: Progress, challenges and recommendations. Nat. Rev. Drug Discov..

[4] Martins H.G.W., Gorski D., Mussa B., Lazo R.E.L., Pontarolo R. (2026). Artificial intelligence in drug discovery for fungal diseases: A scoping review. Artif. Intell. Med..

[5] Antimicrobial Resistance Collaborators (2022). Global burden of bacterial antimicrobial resistance in 2019: A systematic analysis. Lancet.

[6] Stokes J.M., Yang K., Swanson K., Jin W., Cubillos-Ruiz A., Donghia N.M., MacNair C.R., French S., Carfrae L.A., Bloom-Ackermann Z. (2020). A Deep Learning Approach to Antibiotic Discovery. Cell.

[7] El Belghiti I., Hammani O., Moustaoui F., Aghrouch M., Lemkhente Z., Boubrik F., Belmouden A. (2025). Halicin: A New Approach to Antibacterial Therapy, a Promising Avenue for the Post-Antibiotic Era. Antibiotics.

[8] Vamathevan J., Clark D., Czodrowski P., Dunham I., Ferran E., Lee G., Li B., Madabhushi A., Shah P., Spitzer M. (2019). Applications of machine learning in drug discovery and development. Nat. Rev. Drug Discov..

[9] Delcour A.H. (2009). Outer membrane permeability and antibiotic resistance. Biochim. Biophys. Acta.

[10] Liu G., Catacutan D.B., Rathod K., Swanson K., Jin W., Mohammed J.C., Chiappino-Pepe A., Syed S.A., Fragis M., Rachwalski K. (2023). Deep learning-guided discovery of an antibiotic targeting *Acinetobacter baumannii*. Nat. Chem. Biol..

[11] Richter M.F., Drown B.S., Riley A.P., Garcia A., Shirai T., Svec R.L., Hergenrother P.J. (2017). Predictive compound accumulation rules yield a broad-spectrum antibiotic. Nature.

[12] Dara S., Dhamercherla S., Jadav S.S., Babu C.M., Ahsan M.J. (2022). Machine Learning in Drug Discovery: A Review. Artif. Intell. Rev..

[13] Brüssow H. (2024). The antibiotic resistance crisis and the development of new antibiotics. Microb. Biotechnol..

[14] Gutnik D., Evseev P., Miroshnikov K., Shneider M. (2023). Using AlphaFold Predictions in Viral Research. Curr. Issues Mol. Biol..

[15] Zhou Y., Hou Y., Shen J., Huang Y., Martin W., Cheng F. (2020). Network-based drug repurposing for novel coronavirus 2019-nCoV/SARS-CoV-2. Cell Discov..

[16] Sanjuán R., Domingo-Calap P. (2016). Mechanisms of viral mutation. Cell Mol. Life Sci..

[17] Tummino T.A., Rezelj V.V., Fischer B., Fischer A., O’Meara M.J., Monel B., Vallet T., White K.M., Zhang Z., Alon A. (2021). Drug-induced phospholipidosis confounds drug repurposing for SARS-CoV-2. Science.

[18] Bongomin F., Gago S., Oladele R.O., Denning D.W. (2017). Global and Multi-National Prevalence of Fungal Diseases-Estimate Precision. J. Fungi.

[19] Branda F., Petrosillo N., Ceccarelli G., Giovanetti M., De Vito A., Madeddu G., Scarpa F., Ciccozzi M. (2025). Antifungal Agents in the 21st Century: Advances, Challenges, and Future Perspectives. Infect. Dis. Rep..

[20] Perfect J.R. (2017). The antifungal pipeline: A reality check. Nat. Rev. Drug Discov..

[21] Al Shami R., Mousa W.K. (2026). Mining the Hidden Pharmacopeia: Fungal Endophytes, Natural Products, and the Rise of AI-Driven Drug Discovery. Int. J. Mol. Sci..

[22] Yan K., Duan L., Fang W. (2026). Chemical tools to probe antifungal targets. iFungi.

[23] You Z., Zhuang K., Sugita T., Ran Y. (2026). Novel Strategies Against Fungal Infections: Repurposing Existing Drugs and Advancing Nanotherapeutics. iFungi.

[24] Li Y., Qiao Y., Ma Y., Xue P., Ding C. (2025). AI in fungal drug development: Opportunities, challenges, and future outlook. Front. Cell. Infect. Microbiol..

[25] Wu X., Lu J., Ma S., Lin F., Shi X. (2026). Beyond Fungitoxicity: Recent Achievements in Targeted Fungicide Discovery. J. Fungi.

[26] Varadi M., Anyango S., Deshpande M., Nair S., Natassia C., Yordanova G., Yuan D., Stroe O., Wood G., Laydon A. (2022). AlphaFold Protein Structure Database: Massively expanding the structural coverage of protein-sequence space with high-accuracy models. Nucleic Acids Res..

[27] Chai X., Jiang Y., Lu H., Huang X. (2025). Integrating ensemble machine learning and multi-omics approaches to identify Dp44mT as a novel anti-*Candida albicans* agent targeting cellular iron homeostasis. Front. Pharmacol..

[28] Li K., Xia W., Zhang J.Z.H. (2025). Discovery of Novel Inhibitors of *Aspergillus fumigatus* DHODH via Virtual Screening, MD Simulation, and In Vitro Activity Assay. Molecules.

[29] Wang Y., Song M., Liu F., Liang Z., Hong R., Dong Y., Luan H., Fu X., Yuan W., Fang W. (2025). Artificial intelligence using a latent diffusion model enables the generation of diverse and potent antimicrobial peptides. Sci. Adv..

[30] Gow N.A.R., Latge J.P., Munro C.A. (2017). The Fungal Cell Wall: Structure, Biosynthesis, and Function. Microbiol. Spectr..

[31] Pereira R., Dos Santos Fontenelle R.O., de Brito E.H.S., de Morais S.M. (2021). Biofilm of *Candida albicans*: Formation, regulation and resistance. J. Appl. Microbiol..

[32] Le Mauff F. (2020). Exopolysaccharides and Biofilms. Curr. Top. Microbiol. Immunol..

[33] Sudbery P.E. (2011). Growth of *Candida albicans* hyphae. Nat. Rev. Microbiol..

[34] Jumper J., Evans R., Pritzel A., Green T., Figurnov M., Ronneberger O., Tunyasuvunakool K., Bates R., Žídek A., Potapenko A. (2021). Highly accurate protein structure prediction with AlphaFold. Nature.

[35] Zhou Y., Wang Y., Grum-Grzhimaylo A.A., Meijer M., Kraak B., Li Z., Houbraken J. (2025). Contribution of the CYP51A Y119F Mutation to Azole Resistance in *Aspergillus flavus*. J. Fungi.

[36] Subramanian A., Narayan R., Corsello S.M., Peck D.D., Natoli T.E., Lu X., Gould J., Davis J.F., Tubelli A.A., Asiedu J.K. (2017). A Next Generation Connectivity Map: L1000 Platform and the First 1,000,000 Profiles. Cell.

[37] Wu S., Roberts K., Datta S., Du J., Ji Z., Si Y., Soni S., Wang Q., Wei Q., Xiang Y. (2020). Deep learning in clinical natural language processing: A methodical review. J. Am. Med. Inform. Assoc..

[38] Johnson-Arbor K. (2022). Ivermectin: A mini-review. Clin. Toxicol..

[39] Zhu S.P., Wang L., Luo C., Correia J.A.F.O., De Jesus A.M.P., Berto F., Wang Q.Y. (2023). Physics-informed machine learning and its structural integrity applications: State of the art. Philos. Trans. A Math. Phys. Eng. Sci..

[40] Ahmadi N., Cao Q., Humphrey J.D., Karniadakis G.E. (2026). Physics-Informed Machine Learning in Biomedical Science and Engineering. Annu. Rev. Biomed. Eng..

[41] Gao K., Zhang X., Wang W., Li P., Niu S., Bi Y. (2026). Discovery and activity evaluation of fungal chitin synthase inhibitors via virtual screening. Bioorg Chem..

[42] Ingber D.E. (2022). Human organs-on-chips for disease modelling, drug development and personalized medicine. Nat. Rev. Genet..

[43] Secomandi S., Gallo G.R., Rossi R., Rodríguez Fernandes C., Jarvis E.D., Bonisoli-Alquati A., Gianfranceschi L., Formenti G. (2025). Pangenome graphs and their applications in biodiversity genomics. Nat. Genet..

[44] Achilonu C.C. (2026). Targeting Fungal Adaptive Networks and Emerging Molecular Targets for Next-Generation Antifungal Therapeutics. Drugs Drug Candidates.

[45] Li D., Wang Y., Hu W., Chen F., Zhao J., Chen X., Han L. (2021). Application of Machine Learning Classifier to *Candida auris* Drug Resistance Analysis. Front. Cell Infect. Microbiol..

[46] Xiong G., Wu Z., Yi J., Fu L., Yang Z., Hsieh C., Yin M., Zeng X., Wu C., Lu A. (2021). ADMETlab 2.0: An integrated online platform for accurate and comprehensive predictions of ADMET properties. Nucleic Acids Res..

[47] Urbina F., Lentzos F., Invernizzi C., Ekins S. (2022). Dual Use of Artificial Intelligence-powered Drug Discovery. Nat. Mach. Intell..

[48] Qi T., Fang Y., Liu W., He X., Kong L., Chen R., Zhang L. (2026). Applying synthetic biology in infectious disease prevention and facing biosecurity challenges. iFungi.

[49] Ren Y., Li H., Yan W., Liu X., Chi W., Luo R., Stoeger T., Wajid A., Dodovski A., Gao C. (2026). Novel Reassortant H9N2 Avian Influenza Viruses with Dual Receptor-Binding Capacity and Evidence of Direct Mammalian Infectivity Circulating in Northeast China Live Poultry Markets. Viruses.

